# A Grand Challenge in Development and Evodevo: Quantifying the Role of Development in Evolution

**DOI:** 10.3389/fpls.2021.752344

**Published:** 2022-01-11

**Authors:** Aaron R. Leichty, Neelima Roy Sinha

**Affiliations:** Department of Plant Biology, University of California, Davis, Davis, CA, United States

**Keywords:** evodevo, developmental constraints, GRN evolution, morphospace, plasticity-first evolution, plant development

The identification and characterization of genes affecting development and their integration into regulatory networks has been the dominant endeavor over the past 30 years of plant development. The field of evolutionary developmental biology has been greatly aided by these findings, promoting a vast array of comparative work examining how these developmental pathways function in diverse species. However, evodevo as a discipline is not just concerned with how development evolves, but also how development contributes to the process of evolution. Historically, this avenue of study has taken on two major forms—either a focus on how developmental systems constrain or channel evolution in certain directions and not others (Alberch, 1982; Maynard Smith et al., 1985) or how development can promote or lead during evolution, with a particular focus on developmental plasticity and the role of the environment in the production of phenotypic variation (West-Eberhard, 2003). In our view, a grand challenge going forward is for the broader inclusion of these latter questions into the comparative framework that has already been so successfully applied across major morphological transitions of the plant phylogeny. Central to this inclusion will be the focus on well-characterized genetic regulatory networks (GRN) and the evaluation of the phenotypic variation they are capable of producing using ecologically and phylogenetically relevant genetic and environmental manipulations. Together, this focus on the range of phenotypic variation that can be generated and the fitness consequences of such variation will help in quantifying the role of development in generating evolutionary change.

The fact that an organism's developmental systems are incapable of generating phenotypic variation equally in all directions, and therefore impose directionality on the trajectory of evolution, is by now well-accepted (Jablonski, 2020; Salazar-Cuidad, 2021). In plants, work on this concept of developmental constraint (and the inter-related concepts of developmental bias and developmental drive) have been extensive—ranging from studies on the evolution of floral organs (Wessinger and Hileman, 2016) to the role that it may play in structuring defenses to herbivores and pathogens during a plant's life cycle (Boege and Marquis, 2005)—and parallels work done in animals. In some instances, such as flower color, these constraints on trait evolution and their relation to the underlying GRN have been extensively documented (e.g., Rausher, 2008; Smith, 2011; Larter et al., 2018; Ng et al., 2018). However, in many cases of morphological evolution, there remains a great deal of opportunity for defining the gaps in phenotypic potential produced by a particular GRN and to what degree these gaps have coincided with evolutionary trajectories in a phylogenetic context.

Contrary to seeing development as a limiting force in evolutionary change, in recent years research programs have begun to explore how environmentally induced phenotypic variation might actually promote evolution (West-Eberhard, 2003). In particular, this work often centers on the possibility that phenotypic plasticity precedes genetic changes during adaptation—a process now referred to as plasticity-first or plasticity-led evolution (Levis and Pfennig, 2016, 2020). The evidence for this mode of evolution remains small, yet examples in both animals and plants are beginning to accumulate (Bock et al., 2018; Corl et al., 2018). Additionally, the lack of evidence is at least in part a consequence of most instances of putative mutation-first evolution having not been examined for the role that environment may have played in the initial phase of an adaptive event (Wund et al., 2008; Muschick et al., 2011). Further, despite the large number of environmentally regulated phenomena in plants (Olsen, 2019), work examining such developmental events has lagged behind work in animals (Levis and Pfennig, 2020) creating a large potential for new insights into this aspect of developmental evolution.

While the solution to this under-representation must undoubtedly involve increased focus from within the plant community, we believe the way forward is not to directly focus on whether development constrains or promotes evolutionary change (it certainly does both), but to characterize the range and type of phenotypes produced (Salazar-Cuidad, 2006, 2021) by specific GRN through evaluations of ecologically relevant environmental conditions and genetic perturbations within a phylogenetic context. Taking the leaf as an example, multiple GRN responsible for the final form of a leaf have now been described (Chitwood and Sinha, 2016; Conklin et al., 2019) and many of these networks have been characterized across broad phylogenetic scales. For example, it has been shown that the repeated evolution of complex leaves from simple leaves has been mediated by recruitment of KNOXI proteins into networks controlling leaf morphogenesis (Bharathan et al., 2002; Hay and Tsiantis, 2010). Interestingly, more recent work has demonstrated that this same gene regulatory network is responsible for the environmentally induced shifts in leaf morphology of the amphibious plant *Rorippa aquatica*. When this species is grown in a terrestrial environment, simple leaves develop, whereas aquatic conditions produce highly compounded leaves (Fassett, 1930). Further, it was recently found that this difference in leaf morphogenesis is mediated by shifts in KNOXI abundance induced by changes in the light and temperature at which the plants were grown (Nakayama et al., 2014).

In turn, this focus on the GRN underlying leaf complexity, how they are deployed at macroevolutionary scales, and how they are modulated by environmental inputs, opens the door to questions about their role in facilitating the evolutionary trajectories of different lineages. For example, one hypothesis of plasticity-led evolution is that lineages approximating the ancestral species should exhibit developmental plasticity in the trait of interest (Levis and Pfennig, 2016). In the case of *R. aquatica*, this would mean that growing other *Rorippa* species under different light and temperature regimens would be expected to produce phenotypic variation consistent with the alternative phenotypes found in R. *aquatica*. Further, the genetic regulation of these responses will be expected to have undergone refinement in *R. aquatica*, as a result of changes that enhance the association between environmental conditions and the developed phenotype (Levis and Pfennig, 2016). Going forward, we believe that assessing the phenotypic variation of well-characterized GRN under ecologically and phylogenetically relevant conditions will aid in a better understanding of the role development plays in generating evolutionary changes.

Additionally, although constraint has been a major area of focus in plant evodevo we believe that new methods for analysis of phenotypic variation have the potential for informing how GRN features bias the range of phenotypes seen in lineages. In many instances it is helpful to develop a set of phenotypic expectations against which hypothesis of constraint can be tested (i.e., are there phenotypes missing or over represented in a particular group?). One method for generating such hypotheses is the construction of morphospaces, whereby either mathematical models of development or observational data of trait variation can be plotted to infer the potential variation for a set of traits (e.g., Stebbins, 1951; Raup and Michelson, 1965). Within this theoretical or observational space one can hypothesize why certain trait values are rare or abundant. This method has been employed extensively for studies of animal development and is beginning to see more widespread adoption in plants (Chartier et al., 2014; Li et al., 2018), in part aided by methods for the analysis of shape (Chitwood et al., 2016).

Again using leaves as an example, Chitwood and Otoni examined the morphology of leaves across the heteroblastic transition of 40 *Passiflora* species, finding that the earliest leaves produced by each species were more similar across species than leaves produced later in development (Chitwood and Otoni, 2017a,b). When this finding is integrated with what is known about the GRN underlying age-dependent changes in morphology (also known as heteroblasty), a set of testable hypotheses emerge. In *Arabidopsis thaliana*, and many other species, the miR156-SPL pathway is primarily responsible for the heteroblastic transition in leaf morphology (Wu and Poethig, 2006; Wang et al., 2011). Early in development, levels of miR156 are high, but temporal silencing of *MIR156* genes releases *SPL* transcripts from target cleavage or translational repression (Xu et al., 2016). In *A. thaliana* and *Cardamine hirsuta*, heteroblasty is mediated by the competition between SPL and TCP proteins for interaction with CUC proteins which are in turn responsible for production of serrations or leaflets (Blein et al., 2008; Rubio-Somoza et al., 2014). Assuming that this is a general mechanism for increasing complexity during heteroblastic transitions, it would make sense then that *Passiflora* species (and plants more generally) are constrained in their morphological variation early in development when *SPL* genes are repressed by high levels of miR156. More generally the *SPL* gene family may be a node in the GRN that remains relatively unconstrained, thereby allowing for phenotypic divergence between species at later stages of development. Put another way, are the GRN regulating juvenile and adult leaves different in their potential for producing phenotypic variation and does this explain why juvenile morphologies are often conserved within lineages (e.g., Figures 1A,B)? Attempts to experimentally accelerate or alter early leaf morphogenesis would allow for ecological and developmental tests of these possibilities.

**Figure 1:**
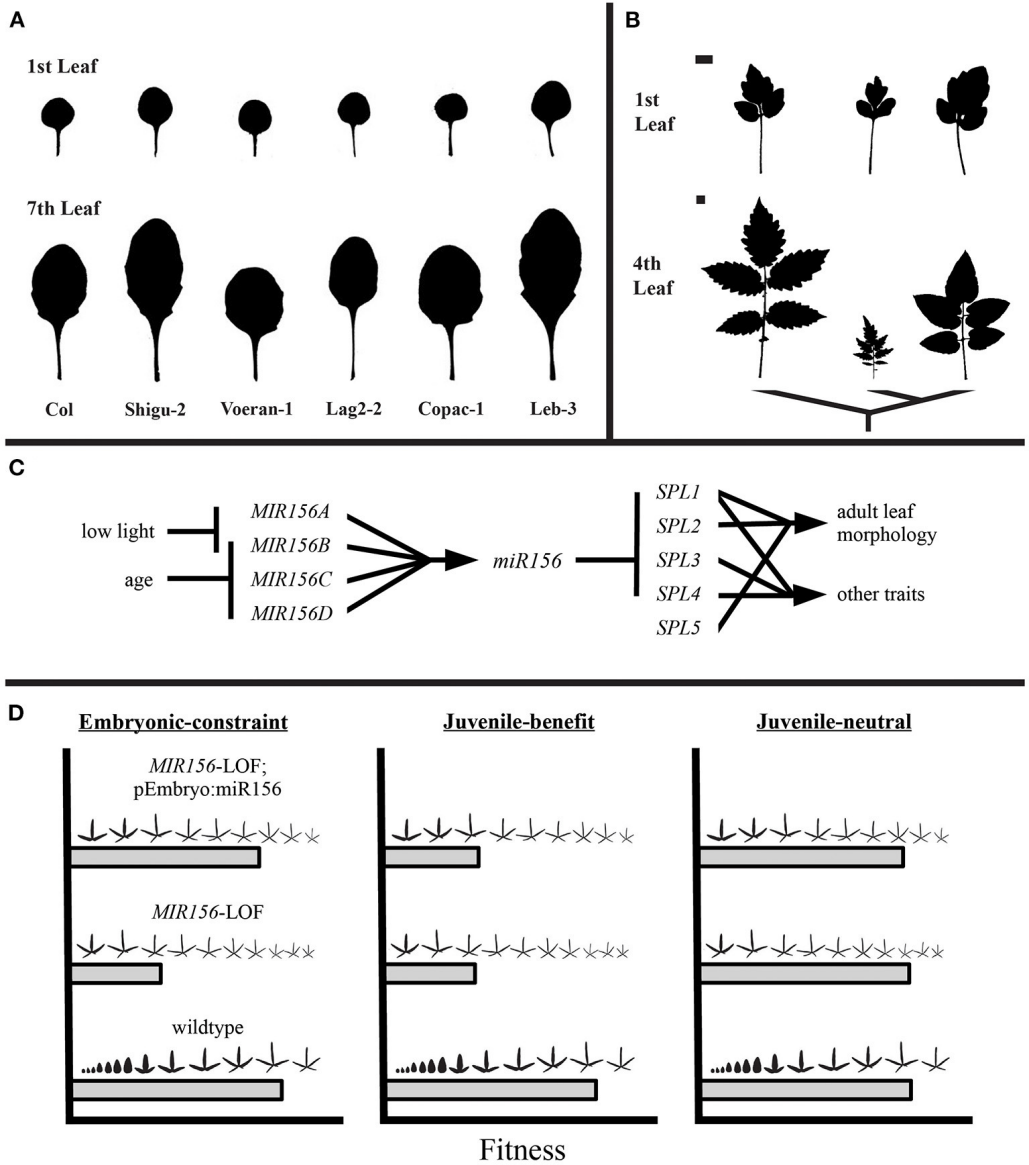
Is there developmental constraint on early leaf morphology? In many clades, the morphology of leaves produced early in the plant life cycle are conserved between species [**(A,B)**, top rows within a panel] relative to leaves produced later [**(A,B)**, bottom rows within a panel]. As the GRN for many aspects of leaf morphogenesis and vegetative transitions are now known, it is theoretically possible to test how development and evolutionary forces such as selection may explain these macroevolutionary patterns. **(A)** Leaves are from *Arabidopsis thaliana* accessions. Adapted from He (2017). **(B)** Leaves from three tomato species. Adapted from Chitwood et al. (2012). Scale bars represent 1 cm and apply for leaves within each row. **(C)** The miR156-SPL pathway in a hypothetical species. **(D)** Predicted outcomes for experimental manipulation of juvenile leaf production and tests of fitness consequences in a hypothetical species. If the need for high levels of miR156 in the embryo ensures that SPL-mediated morphologies cannot be produced early in development, then the fitness of *MIR156* loss-of-function mutants (*MIR156*-LOF) should be rescued by embryo-specific expression of miR156 (left panel). Alternatively, if the phylogenetic conservation of juvenile leaves is due to a common adaptive function across species, then *MIR156*-LOF mutants would be expected to exhibit reduced fitness (middle panel). Conversely, selection on juvenile leaf morphology may be weak or absent, thereby creating minimal patterns of divergence between species (right panel). Leaf heteroblastic series adapted from Chitwood and Otoni (2017a).

In part, the logic of such hypothesis testing rests on the premise that if developmental systems can be manipulated (either environmentally or genetically) to produce phenotypic variation in a new context, then at the very least, a strict developmental constraint can be ruled out. Of course, if such phenotypic variation is experimentally demonstrated, this does not rule out selective constraints that disfavor the appearance of such phenotypic variation. Therefore, these novel phenotypic variants must be scrutinized to pinpoint their underlying tradeoffs. In practice—taking again the example of conserved juvenile leaf morphology across many lineages (Figures 1A,B)—it has been demonstrated that miR156 is necessary for the production of leaves with juvenile morphology. Consequently, the question then becomes what is the performance of plants where the earliest leaves have been manipulated to have an adult morphology? This can be achieved by loss-of-function mutations to key *MIR156* genes (He et al., 2018) which accelerates the production of the adult morphology (Figures 1C,D, *MIR156*-LOF). These precocious mutants could then be evaluated for various components of fitness to better understand their phylogenetic scarcity. For example, in *A. thaliana SPL* genes interfere with proper embryo development (Nodine and Bartel, 2010). Therefore, shifting SPL-mediated phenotypes to earlier stages of development will be constrained by the need for high levels of miR156 in the embryo. Theoretically the embryo defects could be rescued by embryo-specific expression of miR156, allowing for direct quantification of these selective constraints (Figure 1D, *MIR156*-LOF; pEmbryo:miR156). Additionally, alternative explanations for conserved juvenile morphology are that selection favors a common morphology early in development due to conserved functional requirements (e.g., Lawrence et al., 2020) or that selection for divergent phenotypes is weak (Figure 1D, “juvenile-benefit” and “juvenile-neutral,” respectively). Expanding such an analysis across multiple species would then quantify the primary drivers for stasis of juvenile morphology. It should be noted that with all these examples, shifts in morphology are confounded with other biochemical and physiological traits regulated by these same GRN. However, in many cases as more is learned about the molecular genetic mechanisms of a pathway, it may become possible to decouple the multiple traits that it coordinates. For example, once the targets of a particular SPL transcription factor are known, *cis*-elements could be targeted that alter leaf morphogenesis while leaving other biochemical targets intact.

It is exactly these sorts of tests that are required to more fully assess how development limits and leads in the evolution of lineages. Work on flower color has undoubtedly led the way in this regard (Sobel and Streisfeld, 2013), but expanding the study of GRN to include ecologically relevant environmental inputs in combination with knowledge of evolutionary trajectories (derived from phylogenetics and morphometrics) will bring new insights into many new and old model systems. Further, by experimentally manipulating GRN to produce relevant phenotypic variation, it will be possible to test the potential role that selection and development play in determining patterns of trait evolution between species. This work will undoubtedly be aided by new methods for exploring the morphospace of lineages, and by continued development of ecologically relevant model systems.

## Author Contributions

All authors listed have made a substantial, direct, and intellectual contribution to the work and approved it for publication.

## Funding

This work was supported by grants from the USDA NIFA (2014-67013-21700), NSF IOS (1558900), and NSF Postdoctoral Research Fellowship in Biology (IOS-1812043) and Katherine Esau Postdoctoral Fellowship to AL.

## Conflict of Interest

The authors declare that the research was conducted in the absence of any commercial or financial relationships that could be construed as a potential conflict of interest. The handling editor declared a shared affiliation with the authors at time of review.

## Publisher's Note

All claims expressed in this article are solely those of the authors and do not necessarily represent those of their affiliated organizations, or those of the publisher, the editors and the reviewers. Any product that may be evaluated in this article, or claim that may be made by its manufacturer, is not guaranteed or endorsed by the publisher.
